# What need is there for standardization of thyroid uptake or scintigraphy using ^99m^Tc pertechnetate in thyroid disease diagnosis?

**DOI:** 10.1590/S1516-31802002000200002

**Published:** 2002-03-02

The paper by Ramos et al.^1^ that appears in this issue of São Paulo Medical Journal/RPM gives rise to two important questions:

1. Is it necessary to have regional standards for every nuclear medicine examination?
2. Are tests for thyroid uptake or scintigraphy still necessary nowadays?

In addressing the first question, there is an apparent consensus that standardization is necessary, since ethnic, nutritional and geographical variations could be of importance in the metabolism of radiopharmaceuticals used in nuclear medicine examinations. However, how far do we have to go in the standardization process? Does every laboratory have to have its own standards? Can we work with data from very homogeneous or similar regions? Which similarity patterns need to be valued?

I think that most of these questions do not have a simple answer, if only because of the worldwide lack of good standardization studies. What is important is to perform well-controlled experimental studies that may have the capability to confirm and explain epidemiological or anecdotal data in humans. As an example, thyroid uptake of ^99m^Tc pertechnetate, which is used especially in the diagnosis of Plummer's disease, can be influenced by the iodine supply.^2^ Recently, we published an evaluation of ^99m^Tc pertechnetate biodistribution, which became altered in diverse tissues in protein-restricted or energy-restricted rats, both when malnutrition was initiated in adulthood^3^ and in adult rats fed normally whose mothers were malnourished during lactation.^4^ In addition, the use of drugs can interfere with the biodistribution of radiopharmaceuticals in experimental studies.^5^ Thus, there is a need for all the studies that are capable of defining better standards for specific groups of patients, so that nuclear medicine examinations can become a reliable diagnostic tool. This is the case of this study,^1^ in which normal standards for thyroid uptake of ^99m^Tc pertechnetate and scintigraphy in São Paulo are defined.

The other question deals with the usefulness of radioisotope studies in thyroid disease diagnoses. ^131^I, ^123^I and ^99m^Tc pertechnetate are tracers used in both thyroid uptake and scintigraphy. Radioactive iodine is a physiological tracer. ^131^I is still used in most nuclear medicine laboratories in Brazil, but it has the main disadvantages of a long half-life and high energy emission. Since ^123^I was not produced in Brazil until recently, it had a very high cost. We hope that its production at the Nuclear Energy Institute of the National Commission for Nuclear Energy (IEN/CNEN) may decrease its cost. Although ^99m^Tc pertechnetate is not absorbed in the thyroid organ and is therefore not a true tracer of iodine metabolism, its advantages are the lower radiation exposure and the fact that it is readily available in all nuclear medicine laboratories and thus relatively inexpensive.

The current clinical indications for thyroid scintigraphy^6^ include: hyperthyroid patients; euthyroid patients with a solitary nodule or multinodular goiter; patients suspected of having an ectopic thyroid; patients who have had thyroid surgery for cancer. In the case of solitary nodules, the combination of ultrasonography and fine needle aspiration biopsy (FNAB) has gained widespread acceptance for nodular diseases of the thyroid, due to its excellent reliability. Thus, it is preferable to perform FNAB first, and if the result indicates a "follicular neoplasm", scintigraphy may be helpful if the nodule is hyperfunctioning or "hot". This evaluation is very important, since the chance of malignancy in a "hot" nodule is less than 1%. In multinodular goiter, scintigraphy scanning is better for making therapeutic decisions such as surgery or radiotherapy. However, it is only possible to obtain differential imaging diagnoses of substernal masses like goiter through ^123^I thyroid scintigraphy.

Consequently, physicians will remain dependent on thyroid scintigraphy for confirming some thyroid disease diagnoses. The paper by Ramos et al.^1^ contributes to the movement towards standardization and dissemination of this examination, which uses an inexpensive radioactive tracer providing a good quality image.
